# Wnt3a expression is associated with MMP-9 expression in primary tumor and metastatic site in recurrent or stage IV colorectal cancer

**DOI:** 10.1186/1471-2407-14-125

**Published:** 2014-02-24

**Authors:** Myung Ah Lee, Jin-Hee Park, Si Young Rhyu, Seong-Taek Oh, Won-Kyoung Kang, Hee-Na Kim

**Affiliations:** 1 Division of Medical Oncology, Department of Internal Medicine, Cancer Research Institute, College of Medicine, The Catholic University of Korea, Seoul St. Mary’s Hospital, 222 Banpo-daero, Seocho-gu, 137-701 Seoul, Korea; 2 Department of Surgery, Seoul St. Mary’s Hospital, The Catholic University of Korea, Seoul, Korea; 3 Department of Pathology, Seoul Clinical Laboratory Clinic, Seoul, Korea

**Keywords:** Wnt3a, Wnt5a, MMP-9, VEGFR-2, β-catenin, Colorectal cancer

## Abstract

**Background:**

The wnt/β-catenin signaling pathway is known to affect in cancer oncogenesis and progression by interacting with the tumor microenvironment. However, the roles of wnt3a and wnt5a in colorectal cancer (CRC) have not been thoroughly studied. In the present study, we investigated the expression of wnt protein and the concordance rate in primary tumor and metastatic sites in CRC. To determine the relationship of wnt proteins with invasion related protein, we also analyzed the association between wnt protein expression and the expression of matrix metalloproteinase-9 (MMP-9) and vascular endothelial growth factor receptor-2 (VEGFR-2).

**Methods:**

Tumor tissue was obtained from eighty-three paraffin- embedded blocks which were using resected tissue from both the primary tumor and metastatic sites for each patient. We performed immunohistochemical staining for wnt3a, wnt5a, β-catenin, MMP-9 and VEGFR-2.

**Results:**

Wnt3a, wnt5a, β-catenin, and MMP-9 expression was high; the proteins were found in over 50% of the primary tumors, but the prevalence was lower in tissue from metastatic sites. The concordance rates between the primary tumor and metastatic site were 76.2% for wnt5a and 79.4% for wnt3a and β-catenin, but VEGFR-2 was expressed in 67.4% of the metastatic sites even when not found in the primary tumor. Wnt3a expression in primary tumors was significantly associated with lymph node involvement (p = 0.038) and MMP-9 expression in the primary tumor (p = 0.0387), mesenchyme adjacent to tumor (p = 0.022) and metastatic site (p = 0.004). There was no other relationship in the expression of these proteins. Vascular invasion in primary tumor tissue may be a potential prognostic marker for liver metastasis, but no significant association was observed among the wnt protein, MMP-9, and VEGFR-2 for peritoneal seeding. In survival analysis, β-catenin expression was significantly correlated with overall survival (p = 0.05).

**Conclusions:**

Wnt3a and wnt5a expression had a concordance rate higher than 60% with a high concordance rate between the primary tumor and metastatic site. Wnt3a expression is associated with the expression of MMP-9 in primary tumor tissue adjacent mesenchymal tissue, and at the metastatic site. As a prognostic marker, only β-catenin expression showed significant relation with survival outcome.

## Background

Colorectal cancer (CRC) is the 3^rd^ most common cancer in Korea and is becoming more common in Asia and Western countries [1]. As new anti-cancer agents and new technology for local treatment have been developed, the survival rates of patients with CRC have been increasing, even for patients with stage IV cancer. However, distant organ metastasis eventually develops in stage IV cancer, and leads to fatal organ failure. To improve survival outcomes, it is important to control cancer invasion and metastasis, but the mechanisms by which CRC becomes metastasis are not yet known.

The wnt/β-catenin pathway is known to play an important role in maintaining cell homeostasis in normal cells and in embryologic cell development. In CRC, mutation in APC or β-catenin, component of the wnt signaling pathway, are well-known oncogenic factors in familial and some sporadic CRC cases. Recently, many researchers have suggested that the wnt signaling pathway is also involved in controlling cancer cell invasion and metastasis by interacting with the tumor microenvironment or other signal pathways [2-4]. However, most studies investigating the effects of the wnt pathway on CRC have focused on β-catenin, and the role of wnt3a (the initiator of wnt/β-catenin pathway) is not well understood.

The non-canonical pathway via wnt5a is another of the identified wnt signaling pathways. The non-canonical pathway plays a role in embryonic cell motility, but the role of the non-canonical pathway in cancer is unknown. Katoh has suggested that the non-canonical pathway is involved in tumor cell invasion and metastasis [5], but studies investigating the role of wnt5a expression in cancer have been limited and controversial. Several authors have reported that the wnt5a expression is associated with higher grade, poor differentiation or poor clinical outcomes, but others have suggested that wnt5a expression antagonizes the wnt/β-catenin pathway and inhibits oncogenesis [6]. Dejimek et al. suggested that wnt5a expression in stage II colon cancer is associated with good prognosis, and another study reported that wnt5a methylation is associated with microsatellite instability and BRAF mutation [7,8]. Considering these data, the exact role of wnt5a in cancer is unclear.

MMP-9 and VEGF expression are commonly studied in cancer research, and their expression is associated with invasion and metastasis. One study reported that in vitro inhibition of DKK-1 signaling inhibits the MMP-9 expression [9], but MMP-9 expression decreased after wnt3a treatment in another study [10]. Many studies investigating the role of MMP-9 in CRC have been conducted using cell lines or animal models. Few trials have reported that MMP-9 expression can be used as a prognostic factor for disease recurrence in human tissue [11]. Thus, the association between the wnt signaling pathway and MMP-9 and VEGFR-2 expression in CRC remains unclear.

Previous experiments investigating the role of the wnt proteins in metastasis have been conducted at the primary site of stages I-III CRC because diseased tissue can be easily obtained through surgery. Studies focusing on the relationships between the primary tissue and tissue from the metastatic site have rarely been reported. Recently, resection of metastatic lesions has become a treatment option for patients with stage IV CRC. In the present study, we evaluated the expression of wnt3a, wnt5a, MMP-9, and VEGFR-2 in human tissue from primary and metastatic sites of stage IV advanced CRC patients to identify associations between these proteins. We also analyzed the concordance between the primary and metastatic lesions, and aimed to identify the potential prognostic markers of survival outcome.

## Methods

### Patients

This study included eighty-three patients with colon or rectal cancer who had resection for both a primary mass and metastatic lesions resected in a single procedure at Seoul St. Mary’s Hospital between January 2000 and December 2006. All patients had colorectal cancer with organ metastasis at the initial diagnosis. Clinical records and pathological reports were reviewed retrospectively. This study was approved by the institutional review board of Seoul St. Mary’s hospital (KC10SIMI0621).

### Tissue microarray (TMA) and immunohistochemical staining

Core biopsies with 3.0 mm diameter were taken from representative areas of tumor tissue. Each patient had biopsy samples taken from the primary tumor mass and its adjacent mesenchyme as well as the metastatic site and its adjacent mesenchyme. Tissue cores from each specimen were assembled on a recipient paraffin block with a precision instrument (Micro Digital Co. Korea), following previously established methods [12].

We performed immunohistochemical staining on 5 μm sections of the TMA blocks. Each paraffin section was deparaffinized for 1 hour at 60°C in xylene and rehydrated in serial graded ethanol before being stored overnight in citrate buffer (0.01 M, pH 6.0) at 75°C for antigen retrieval. Endogenous peroxidase activity was blocked with 0.3% hydrogen peroxide in methanol. Sections were incubated for 1 hour at room temperature with the following primary antibodies at the specified dilutions: Wnt3a (Abcam, Cambridge, UK) diluted 1:100, Wnt5a (Abcam, Cambridge, UK) diluted 1:50, β-catenin (Abcam, Cambridge, UK) diluted 1:100, MMP-9 (Cell Signaling, Danvers, MA, USA) diluted 1:100, and VEGFR-2 (Cell Signaling, Danvers, MA, USA) diluted 1:200. Immunohistochemical staining was performed using the rabbit or mouse DAKO ChemMate TM EnVision TM system and a Peroxidase/DAB kit (DAKO). Sections were then counterstained with Mayer hematoxylin and dehydrated, cleared and mounted.

**Figure 1:**
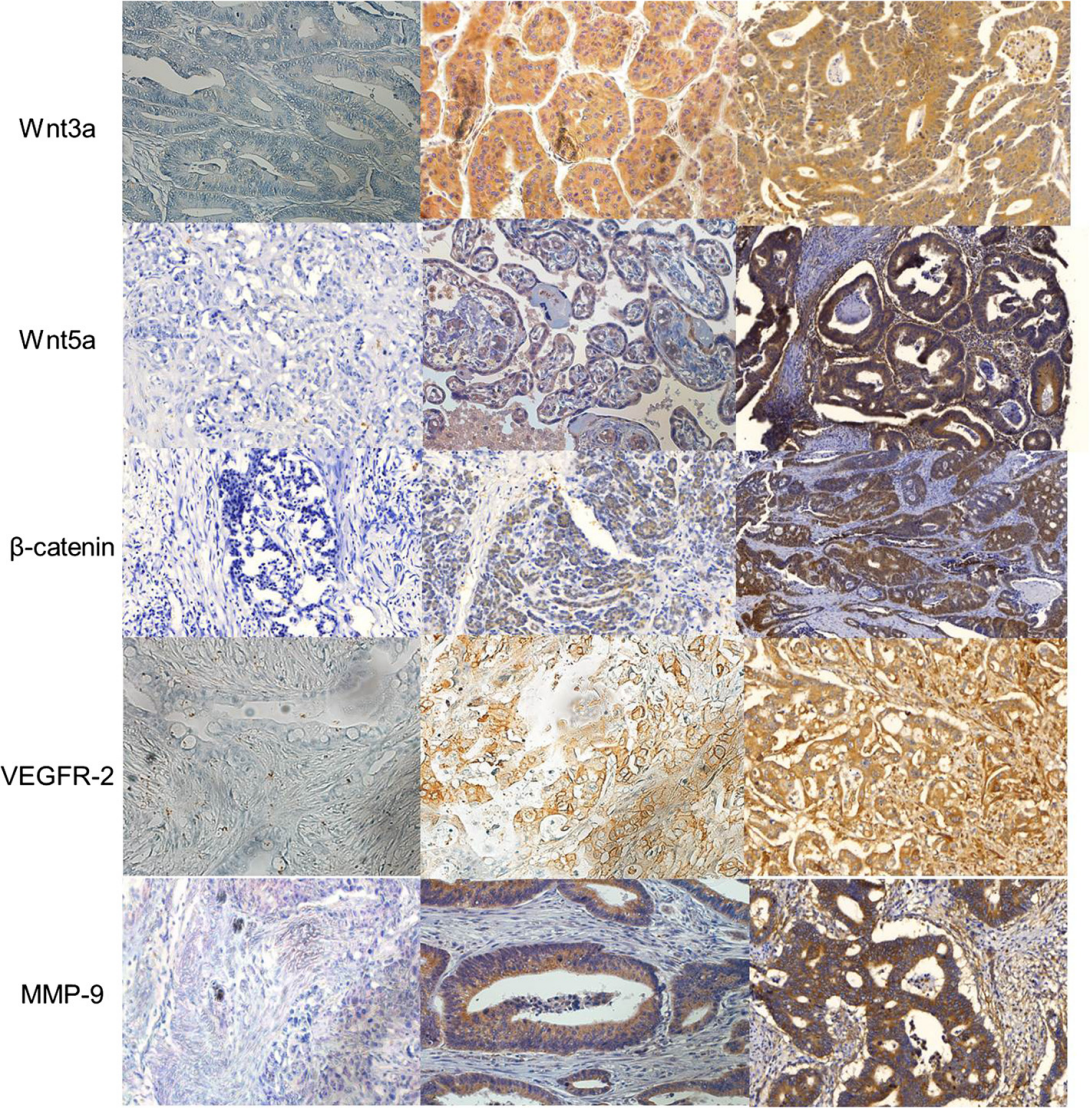
Immunohistochemical staining for the wnt3a, wnt5a, β-catenin, MMP-9, and VEGFR-2 (magnification x 200).

The results were interpreted by two independent pathologists who were blinded to the specific diagnosis and prognosis for each case. The staining intensity was scored on a three-tiered scale: score 0 = less than 10% of cells positive; 1 = 10–49% positive; and 2 = more than 50% of cells positive. The criterion for positive staining was more than 1+ of tumor cells that showed distinct nuclear or cytoplasmic staining (Figure 1).

### Statistical analysis

Continuous and categorical variables were compared using the Student’s *t* test and chi-square test. All statistical analyses were performed using SPSS (version 13.0) and p value under 0.05 was considered statistically significant.

## Results

### Patient characteristics and protein expression

Of the 83 patients, 46 were male and the median age was 60 years. Liver metastasis was the most common (57.8%), followed by peritoneum metastasis (26.5%). Thirteen patients had no lymph node involvement even with stage IV disease, and two patients had T2 disease. The patient characteristics are summarized in Table 1.

**Table 1:** Patients’ characteristics

		**Number**
Age	Years	60 (27–78)
Sex	M : F	46 : 37
Location	Colon	51 (61.4%)
	Rectum	32 (38.6%)
T	2	2 (2.4%)
	3	24 (28.9%)
	4	57 (68.7%)
N	0	13 (15.7%)
	1	26 (31.3%)
	2	44 (53.0%)
Metastasis	Liver	48 (57.8%)
	Peritoneum	22 (26.5%)
	Lung	4 (4.8%)
	Ovary	3 (3.6%)
	Others	6 (7.2%)

Wnt3a, wnt5a, MMP-9 and β-catenin were expressed in more than 50% of the primary tumors, but VEGFR-2 was not. These protein expression levels were slightly decreased in the tissue taken from the metastatic sites (Table 2). We analyzed the concordance rate of the protein expression between the primary tumor and metastatic site. The concordance rates of wnt3a, wnt5a and β-catenin expression were high; all of the rates were in the range of 76.2% to 79.4%, and MMP-9 expression had a 68.3% concordance rate. However, VEGFR-2 was expressed in 67.4% of the metastatic sites when there was no expression in the primary tumors, with only a 40.0% concordance rate between primary tumor and metastatic sites.

**Table 2:** Immunohistochemical staining for primary tumors and metastatic site

	**Wnt3a**	**Wnt5a**	**β-catenin**	**MMP-9**	**VEGFR-2**
Primary tumor	51 (61.4%)	58 (69.9%)	70 (84.3%)	45 (54.2%)	30 (36.1%)
Metastatic site	38 (45.8%)	39 (47.0%)	44 (53.0%)	28 (33.7%)	22 (26.5%)
Concordance rate	79.4%	76.2%	79.4%	68.3%	40.0%

### Wnt3a expression in primary tumor correlated with lymph node involvement and MMP-P expression

We analyzed the association between wnt expression in the primary tumor and the clinicopathologic findings, including the T & N stage. Wnt3a expression in the primary tumor was significantly correlated with lymph node involvement (p = 0.038) and MMP-9 expression in primary, adjacent mesenchyme and metastatic sites (p = 0.038, 0.022 and 0.004, respectively). There was no association between wnt5a expression and other findings, but wnt5a expression did show a correlation tendency with lymph node and lymphatic invasion. This result is summarized in Table 3.

**Table 3:** The association of wnt expression in primary tumor and other pathologic findings

		**Wnt3a**		**Wnt5a**	
		**No.**	**p**	**No.**	**p**
T	2	1		0	
	3	13	0.626	17	*0.092*
	4	37		41	
N	0	8		9	
	1	21	0.038	19	0.910
	2	22		30	
Grade	Well	6		6	
	Moderate	37	0.268	45	0.945
	Poorly	8		7	
Lymphatic invasion	(+)	46	0.482	54	*0.088*
Venous invasion	(+)	16	0.594	20	0.249
Perineural invasion	(+)	34	0.060	37	0.136
Primary tumor	β-catenin	46	0.063	52	0.054
	MMP-9	34	0.004	33	0.306
	VEGFR-2	21	0.166	24	0.102
Adjacent	β-catenin	39	0.299	37	0.289
Mesemchyme	MMP-9	32	0.022	32	0.359
	VEGFR-2	15	0.205	17	0.158
Metastatic site	β-catenin	29	0.382	32	0.598
	MMP-9	23	0.031	20	0.511
	VEGFR-2	16	0.396	15	0.364

Analysis of liver or peritoneal metastasis, wnt expression, MMP expression, and VEGFR-2 expression in the primary tumor did not show any associations but venous invasion was associated with liver metastasis (p = 0.047). We also performed immunohistochemical staining of the adjacent mesenchymal tissue, but there was no association between wnt3a, wnt5a, MMP, or VEGFR-2 expression and liver or peritoneal metastasis (Table 4).

**Table 4:** The association between the protein expression and liver or peritoneal seeding

		**Liver**		**Peritoneum**	
		**No.**	**p**	**No.**	**p**
T	2	1		0	
	3	18	0.130	4	0.264
	4	29		18	
N	0	6		2	
	1	17	0.508	5	0.243
	2	25		15	
Grade	Well	4		3	
	Moderate	39	0.749	17	0.419
	Poorly	5		2	
Lymphatic invasion	(+)	44	0.304	20	0.556
Venous invasion	(+)	19	0.047	5	0.230
Perineural invasion	(+)	27	0.353	15	0.223
Primary tumor	Wnt3a	30	0.498	13	0.493
	Wnt5a	34	0.506	12	0.062
	β-catenin	39	0.277	18	0.470
	MMP-9	26	0.584	10	0.238
	VEGFR-2	15	0.196	9	0.385
Adjacent mesenchyme	Wnt3a	18	0.498	11	0.151
	Wnt5a	29	0.298	15	0.412
	β-catenin	36	0.453	15	0.347
	MMP-9	25	0.510	12	0.533
	VEGFR-2	13	0.431	5	0.494

**Figure 2:**
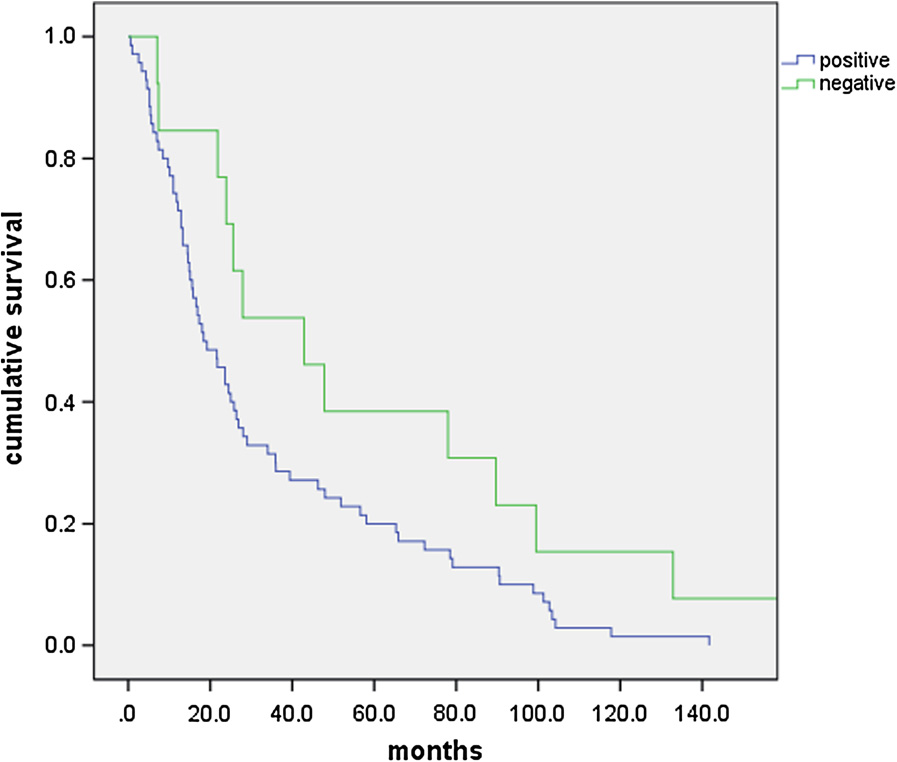
**Overall survival according to β-catenin expression.** β-catenin-expressing group in primary tumor showed poorer survival outcome than non-expressing group (18.4 vs. 42.9 months, p = 0.05).

In survival analysis, patients with positive β-catenin staining in primary tumors showed significantly poorer survival outcomes than those with no staining (18.4 months vs. 42.9 months, respectively, p = 0.05, Figure 2). There were no other prognostic factors for survival.

## Discussion

The wnt/β-catenin signaling pathway is an emerging target for cancer research; studies indication the pathway may play a role in cancer invasion and progression by interacting with the tumor microenvironment and oncogenesis [13]. Wnt/β-catenin has been widely studied as a prognostic factor for CRC, and it may also be involved in the mechanism of cancer invasion [14,15]. However, research has focused on aberrant nuclear β-catenin rather than wnt3a, despite the role of wnt3a as a major initiating factor in the wnt/β-catenin pathway. In the present study, we analyzed wnt3a and wnt5a to determine their roles in cancer progression. We selected the patients who had had both their primary tumor and the metastatic sites resected to identify differences in the protein expression between the primary and the metastatic sites. Expression of wnt3a was very high in tissues from both primary tumors and metastatic sites but was higher at the primary site with a concordance rate higher than 70%. Wnt expression at the metastatic site was rare if the primary tumor tested negative for both wnt3a and wnt5a. This result suggests that wnt could be expressed initially when CRC develops, not newly emerged as the cancer progresses. This result is consistent with the previous studies on the oncogenic role of the wnt signaling pathway in various cancers [16,17]. Recently, Boutros reported that sustained wnt activity through wnt3a and Evi/Wls/GPR177 can be important for the proliferation in colon cancer cell, independently from APC or β-catenin mutation [18]. It suggests that the upper stream factor of wnt signaling pathway may play an important role in the cancer progression.

It is well understood that MMP-9 overexpression is a key factor in degradation of the extracellular matrix, an essential step in tumor invasion and metastasis; this role has been observed in human tissue and cell line studies of CRC [19,20]. In the present study, wnt3a expression was significantly correlated with MMP-9 expression in the primary tumor, mesenchyme and metastatic site. In previous study, inhibition of the wnt/β-catenin pathway decreased the level of MMP-9 mRNA in embryonic neural stem cells [9]. These data suggest that the wnt/β-catenin signaling pathway may have an effect on MMP-9 expression, with a role in cancer invasion and metastasis. However, in one mouse study, wnt3a stimulation was shown to inhibit MMP-2 and MMP-9 expression in mesenchymal stem cells; the investigator suggested that regulation of wnt3a could be different in mice and human [13]. Recently, some studies have reported that inhibition of β-catenin by some agents can also inhibit MMP-2 or MMP-9 expression [21,22]. The previous data suggest that the wnt/β-catenin pathway may play a role in cancer invasion and metastasis through MMP-9 expression.

VEGFR is also a topic of interest in cancer proliferation and metastasis research. Many studies have associated the wnt/β-catenin signaling pathway with VEGFR activity [23,24]. In this study, however, wnt expression was not correlated with VEGFR-2 expression. In addition, VEGFR-2 expression was relatively low in our study, particularly at the metastatic site. In other experiments, VEGFR-1 expression was regulated independent of the wnt/β-catenin pathway, and VEGFR-2 did not show any significant association with lymph node or lymphovascular invasion [25,26]. Based on these data, the mechanism by which VEGFR is involved in metastasis should be explored independently of the wnt signaling pathway.

Wnt5a is a key initiating factor in the non-canonical pathway, but its role in cancer is not known. Kato has suggested that the non-canonical pathway may be involved in cancer cell invasion [5]. In previous study, wnt5a expression showed aggressive behavior in breast or gastric cancer [27,28]. However, wnt5a has also been associated with a good prognosis or tumor suppression by inhibiting the wnt/β-catenin pathway in CRC [7,29]. In our study, wnt5a showed no correlation with pathologic findings or invasion related protein expression, but showed higher expression in the primary and metastatic tumor sites. The data do not show an antagonistic relationship between wn3a and wnt5a in the present study. To determine the role of wnt5a in CRC, further analysis of other signaling pathways is warranted.

Theoretically, wnt3a expression is directly associated with β-catenin expression. However, previous studies have reported that β-catenin can be independently, aberrantly expressed without altering wnt3a in CRC [14,15] and could not be differentiated from the β-catenin that is activated by wnt3a. This is the reason β-catenin was higher than wnt3a expression in our study. In survival analysis of our study, β-catenin expression was significantly correlated with poor survival outcome, independently of wnt3a expression. It has previously been shown that the β-catenin expression can be independent prognostic marker for CRC patients.

As a prognostic factor for overall survival, β-catenin expression was significantly correlated only with the survival outcome. Known prognostic factors, such as lymph node involvement or lymphovascular invasion, did not show any significance in our survival analysis. We analyzed the stage IV patients with metastasis in the present study; these factors could have less of effect on the survival status in stage IV patients than in stage II or III CRC patients.

There is a limitation in our study. We could not determine whether the wnt and MMP-9 expression levels are prognostic or predictive factors because we performed the present study in stage IV CRC patients. According to the objective of this study, we enrolled the patients who underwent surgery for primary and metastatic sites; thus, patients with early stages of CRC were not included. Therefore, a comparative study would be required to determine whether wnt and MMP-9 expression levels are prognostic factors for the recurrence of distant metastasis.

In summary, wnt3a and wnt5a expression is high in primary and metastatic tumors in CRC with a high concordance rate. The wnt3a expression is highly correlated with MMP-9 expression, but not with VEGFR-2, and we did not determine the role of wnt5a. To investigate the mechanism of invasion and metastasis, further studies of the wnt/β-catenin pathway and MMP-9 should be performed, and another approach for evaluating VEGFR or wnt5a should be explored.

## Conclusions

Wnt3a and wnt5a are highly expressed in colorectal cancer both in primary and metastatic sites with a higher than 50% concordance rate. The wnt3a expression is significantly associated with MMP-9 expression, the metastasis related protein, but is not related with VEGFR-2 expression, and other metastatic related protein.

## Abbreviations

CRC: Colorectal cancer; MMP-9: Matrix metalloprroteinase-9; TMA: Tissue microarray; VEGF: Vascular endothelial growth factor; VEGFR: Vascular endothelial growth factor receptor.

## Competing interests

The authors declare that they have no competing interests.

## Authors’ contributions

MAL suggested the idea and designed the all research process, took part in acquision of clinical data, analyzed & interpreted of all the data, finally drafted & revised the manuscript. JHP performed the experimental, analyzed the data and reviewed the manuscript. SYR was in charge of collecting all the clinical data, analyed and interpreted the data. STO & WKK supplied all the tissue specimen, collected and interpreted data, and reviewed & commented the manuscript. HNK interpreted the pathological findings, immunohistochemical staining, reviewed & commented the manuscript. All authors read and approved the final manuscript.

## Pre-publication history

The pre-publication history for this paper can be accessed here:

http://www.biomedcentral.com/1471-2407/14/125/prepub

## References

[B1] Jung KW Park SH Won YJ Kong YJ Lee JY Park EC Lee JS Prediction of cancer incidence and mortality in Korea Cancer Res Treat 2011 43 1 12 18 10.4143/crt.2011.43.1.12 21509158 PMC3072530

[B2] Polarkis P Wnt signaling and cancer Genes Dev 2000 14 1837 1851 10921899

[B3] Saif MW Chu E Biology of colorectal cancer Cancer J 2010 16 3 196 201 10.1097/PPO.0b013e3181e076af 20526096

[B4] Huang D Du X Crosstalk between tumor cells and microenvironment via wnt pathway in colorectal cancer dissemination J Gastroenterol 2008 14 12 1823 1827 10.3748/wjg.14.1823 PMC2700405 18350618

[B5] Katoh M WNT/PCP signaling pathway and human cancer (review) Oncol Repub 2005 14 6 1583 1588 16273260

[B6] McDonald SL Silver A The opposing roles of wnt-5a in cancer Br J Can 2009 101 209 214 10.1038/sj.bjc.6605174 PMC2720208 19603030

[B7] Dejimek J Dejimek A Säfholm A Sjölander A Andersson T Wnt-5a protein expression in primary dukes B colon cancers identifies a subgroup of patients with good prognosis Cancer Res 2005 65 20 9142 9146 10.1158/0008-5472.CAN-05-1710 16230369

[B8] Rawson JB Mrkonjic M Daftary D Dicks E Buchanan DD Younghusband HB Parfrey PS Young JP Pollett A Green RC Gallinger S McLaughlin JR Knight JA Bapat B Promoter methylation of Wnt5a is associated with microsatellite instability and BRAF V600E mutation in two large populations of colorectal cancer patients Br J Cancer 2011 104 12 1906 1912 10.1038/bjc.2011.165 21587258 PMC3111198

[B9] Ingraham CA Park GC Makarenkova HP Crossin K Matrix Metalloproteinase (MMP)-9 induced by wnt signaling increases the proliferation and migration of embryonic neural stem cells at low O2 levels J Bio Chem 2011 286 20 17649 17657 10.1074/jbc.M111.229427 21460212 PMC3093840

[B10] Karow M Popp T Egea V Ries C Jochum M Neth P Wnt signaling in muse mesenchymal stem cells: impact on proliferation, invasion and MMP expression J Cell Mol Med 2009 13 88 2506 2520 19413884 10.1111/j.1582-4934.2008.00619.x PMC6529961

[B11] Bendardaf R Buhmeida A Hilska M Laato M Syrjänen S Syrjänen K Collan Y Pyrhönen S MMP-9 (gelatinase B) expression is associated with disease-free survival and disease-specific survival in colorectal cancer patients Cancer Invest 2010 28 1 38 43 10.3109/07357900802672761 20001295

[B12] Lee MA Park KS Lee HJ Jung JH Kang JH Hong YS Lee KS Kim DG Kim SN Survivin expression and its clinical significance in pancreatic cancer BMC Cancer 2005 5 127 132 10.1186/1471-2407-5-127 16202147 PMC1266027

[B13] Neth P Ciccarella M Egea V Hoelters J Jochum M Ries C Wnt signaling regulates the invasion capacity of human mesenchymal stem cells Stem Cells 2006 24 8 1892 1903 10.1634/stemcells.2005-0503 16690780

[B14] Elzagheid A Buhmeida A Korkeila E Collan Y Syrjanen K Pyrhonen S Nuclear beta-catenin expression as a prognostic factor in advanced colorectal carcinoma World J Gastroenterol 2008 14 24 3866 3871 10.3748/wjg.14.3866 18609711 PMC2721444

[B15] Suzuki H Masuda N Shimura T Araki K Kobayashi T Tsutsumi S Asao T Kuwano H Nuclear beta-catenin expression at the invasive front and in the vessels predicts liver metastasis in colorectal carcinoma Anticancer Res 2008 28 3B 1821 1830 18630466

[B16] Kahlil S Tan GA Giri DD Zou XK Howe LR Activation status of Wnt/β-catenin signaling in normal and neoplastic breast tissues: relationship to HER2/neu expression in human and mouse PLos One 2012 7 3 e33421 10.1371/journal.pone.0033421 22457761 PMC3311643

[B17] Moyes LH McEwan H Radulescu S Pawlikowski J Lamm CG Nixon C Sansom OJ Going JJ Fullarton GM Adams PD Activation of Wnt signalling promotes development of dysplasia in Barrett's oesophagus J Pathol 2012 228 1 99 112 22653845 10.1002/path.4058

[B18] Voloshanenko O Erdmann G Dubashi TD Augustin I Metzig M Moffa G Hundsrucker C Ken G Sandmann T Anchang B Demir K Boehm C Leible S Ball CR Glimm H Spang R Boutros M Wnt secretion is required to maintain high levels of wnt activity in colon cancer cells Nat Commun 2013 4 2610 24162018 10.1038/ncomms3610 PMC3826636

[B19] Cheung LW Leung PC Wong AS Gonadotropin-releasing hormone promotes ovarian cancer cell invasiveness through c-Jun NH2-terminal kinase-mediated activation of matrix metalloproteinase (MMP)-2 and MMP-9 Cancer Res 2006 66 22 10902 10910 10.1158/0008-5472.CAN-06-2217 17108127

[B20] Roh SA Choi EY Cho DH Jang SJ Kim SY Kim YS Kim JC Growth and invasion of sporadic colorectal adenocarcinomas in terms of genetic change J Korean Med Sci 2010 25 353 360 20191032 10.3346/jkms.2010.25.3.353 PMC2826746

[B21] Song KS Li G Kim JS Jing K Kim TD Kim JP Seo SB Yoo JK Park HD Hwang BD Lim K Yoon WH Protein-bound polysaccharide from Phellinus linteus inhibits tumor growth, invasion, and angiogenesis and alters Wnt/β-catenin in SW480 human colon cancer cells BMC Cancer 2011 11 307 10.1186/1471-2407-11-307 21781302 PMC3154178

[B22] Singh T Katiyar SK Honokiol Inhibits Non-Small Cell Lung Cancer Cell Migration by Targeting PGE2-Mediated Activation of β-Catenin Signaling PLos One 2013 8 3 e60749 23580348 10.1371/journal.pone.0060749 PMC3620279

[B23] Naik S Dotharger RS Marasa J Lewis CL Piwnica-Worms D Vascular endothelial growth factor receptor-1 is synthetic lethal to aberrant beta catenin activation in colon cancer Clin Cancer Res 2009 15 24 7529 7537 10.1158/1078-0432.CCR-09-0336 20008853 PMC2797340

[B24] Zeitlin BD Ellis LM Nor JE Inhibition of vascular endothelial growth factor receptor-1/wnt beta catenin cross talk leads to tumor cell death Clin Cancer Res 2009 15 24 7453 7455 10.1158/1078-0432.CCR-09-2578 20008844 PMC2796551

[B25] Yoshihara T Takahashi-Yanaga F Shiraishi F Morimoto S Watanabe Y Hirata M Hoka S Sasaguri T Anti-angiogenic effects of differentiation-inducing factor-1 involving VEGFR-2 expression inhibition independent of the Wnt/β-catenin signaling pathway Mol Cancer 2010 16 9 245 20843378 10.1186/1476-4598-9-245 PMC2946290

[B26] Kin JU Bae BN Kim HJ Park KM Prognostic significance of epidermal growth factor receptor and vascular endothelial growth factor receptor in colorectal adenocarcinoma APMIS 2011 119 7 449 459 10.1111/j.1600-0463.2011.02752.x 21635552

[B27] Kurayoshi M Oue N Yamamoto H Kishida M Inoue A Asahara T Yasui W Kikuchi A Expression of Wnt-5a is correlated with aggressiveness of gastric cancer by stimulating cell migration and invasion Cancer Res 2006 66 21 10439 10448 10.1158/0008-5472.CAN-06-2359 17079465

[B28] Pukrop T Klemm F Hagemann T Gradl D Schulz M Siemes S Trümper L Binder C Wnt5a signaling is critical for macrophage-induced invasion of breast cancer cell lines Proc Natl Acad Sci U S A 2006 103 14 5454 5459 10.1073/pnas.0509703103 16569699 PMC1459376

[B29] Ying J Li H Yu J Ng KM Poon FF Wong SC Chan AT Sung JJ Tao Q WNT5A exhibits tumor-suppressive activity through antagonizing the Wnt/beta-catenin signaling, and is frequently methylated in colorectal cancer Clin Cancer Res 2008 14 1 55 61 10.1158/1078-0432.CCR-07-1644 18172252

